# Efficacy of ripasudil-brimonidine fixed-dose combination in secondary glaucoma associated with uveitis: a retrospective study

**DOI:** 10.1007/s10384-026-01361-0

**Published:** 2026-04-22

**Authors:** Kenji Miyao, Masaya Imazeki, Tomoyuki Oyama, Shunsaku Nakai, Takayuki Kanda, Masaru Takeuchi

**Affiliations:** Department of Ophthalmology, National Defense Medical College, 3-2 Namiki, Tokorozawa, Saitama 359-8513 Japan

**Keywords:** Brimonidine, Ocular hypertension, Ripasudil, Secondary glaucoma, Uveitis

## Abstract

**Purpose:**

To evaluate the intraocular pressure (IOP)-lowering efficacy and treatment stability of ripasudil-brimonidine fixed-dose combination (RBFC) in patients with secondary glaucoma associated with uveitis.

**Study design:**

Single-center, retrospective study.

**Methods:**

Medical records of 50 eyes prescribed RBFC were reviewed and categorized into inflammatory ocular hypertension (OH; *n* = 19), steroid-induced OH (*n* = 18), and primary open-angle glaucoma (POAG; *n* = 13). Clinical parameters—including IOP, visual acuity (VA), and medication score—were assessed at baseline, the first follow-up, and the final visit.

**Results:**

In the inflammatory and steroid-induced OH groups, mean IOP decreased significantly from baseline and remained stable throughout the observation period (*P* < 0.001). VA was maintained with a slight, non-significant improvement, and medication scores remained stable, indicating sustained treatment efficacy without additional eye drops. One mild case of conjunctivitis occurred in the POAG group.

**Conclusion:**

RBFC was associated with significant IOP reduction in eyes with uveitis-associated or steroid-induced OH, without compromising visual function. These findings suggest that RBFC may be a useful therapeutic option for inflammation-related secondary glaucoma.

## Introduction

Uveitis refers to a group of intraocular inflammatory disorders triggered by diverse etiologies such as infection, autoimmune dysfunction, or ocular trauma [1]. It is a significant contributor to global visual loss, accounting for approximately 10–15% of cases of blindness and causing severe or legal visual impairment in a large proportion of affected individuals [2, 3]. Glaucoma secondary to uveitis is a sight-threatening complication [4, 5], and 10–20% of patients with uveitis will develop secondary glaucoma over the disease course. Since intraocular pressure (IOP) higher than 30 mmHg is not rare, most patients require multiple antiglaucoma medications [6–8].

Ripasudil hydrochloride hydrate is a Rho-kinase (ROCK) inhibitor that improves aqueous humor drainage through the trabecular outflow pathway [9–11]. It reduces actomyosin contraction, disrupts stress fibers in trabecular meshwork cells, and enhances the permeability of the inner wall of Schlemm’s canal. These effects decrease outflow resistance and consequently lower IOP. Beyond biomechanics, ROCK inhibition also exerts immunomodulatory effects relevant to uveitis, including dampening leukocyte trafficking and pro-inflammatory cytokine signaling; in experimental autoimmune uveoretinitis and endotoxin-induced uveitis models, ripasudil reduced clinical inflammation and inflammatory readouts, supporting a potential dual benefit in inflamed eyes [12, 13]. Clinical studies indicate that ripasudil effectively lowers IOP in uveitis-related secondary glaucoma (including inflammatory and steroid-induced types), [14–16], and that its perioperative use contributes to improved surgical success. [17].

Brimonidine tartrate, an α2-adrenergic receptor agonist, lowers IOP via a complementary mechanism—reducing aqueous humor production acutely and enhancing uveoscleral outflow with chronic dosing [18–20]. In addition, it is suggested that brimonidine may confer retinal ganglion cell neuroprotection through NMDA/glutamate pathway modulation and trophic factor signaling, which could be advantageous in eyes at risk of inflammatory optic neuropathy [19, 21].

Recently, ripasudil-brimonidine fixed-dose combination (RBFC) has shown durable IOP-lowering efficacy and acceptable safety in glaucoma/ocular hypertension (OH) [22], and switching studies suggest RBFC achieves greater IOP reduction than either agent alone [23]. Since anti-inflammatory and mydriatic eye drops are generally prescribed for uveitic eyes, antiglaucoma eye drops for secondary glaucoma or OH are usually added as a third medication. Considering issues of compliance and the efficacy of IOP-lowering therapy, fixed-combination eye drops are favorable, and RBFC, which may also provide anti-inflammatory benefits, is even more advantageous. The purpose of this study was to evaluate the IOP–lowering efficacy, treatment stability, and safety of RBFC in patients with uveitis-associated glaucoma, and to compare these outcomes with those in primary open-angle glaucoma (POAG), thereby clarifying the therapeutic value of RBFC in inflammation- or corticosteroid-related IOP elevation where treatment options remain limited.

## Methods

**Fig 1.**
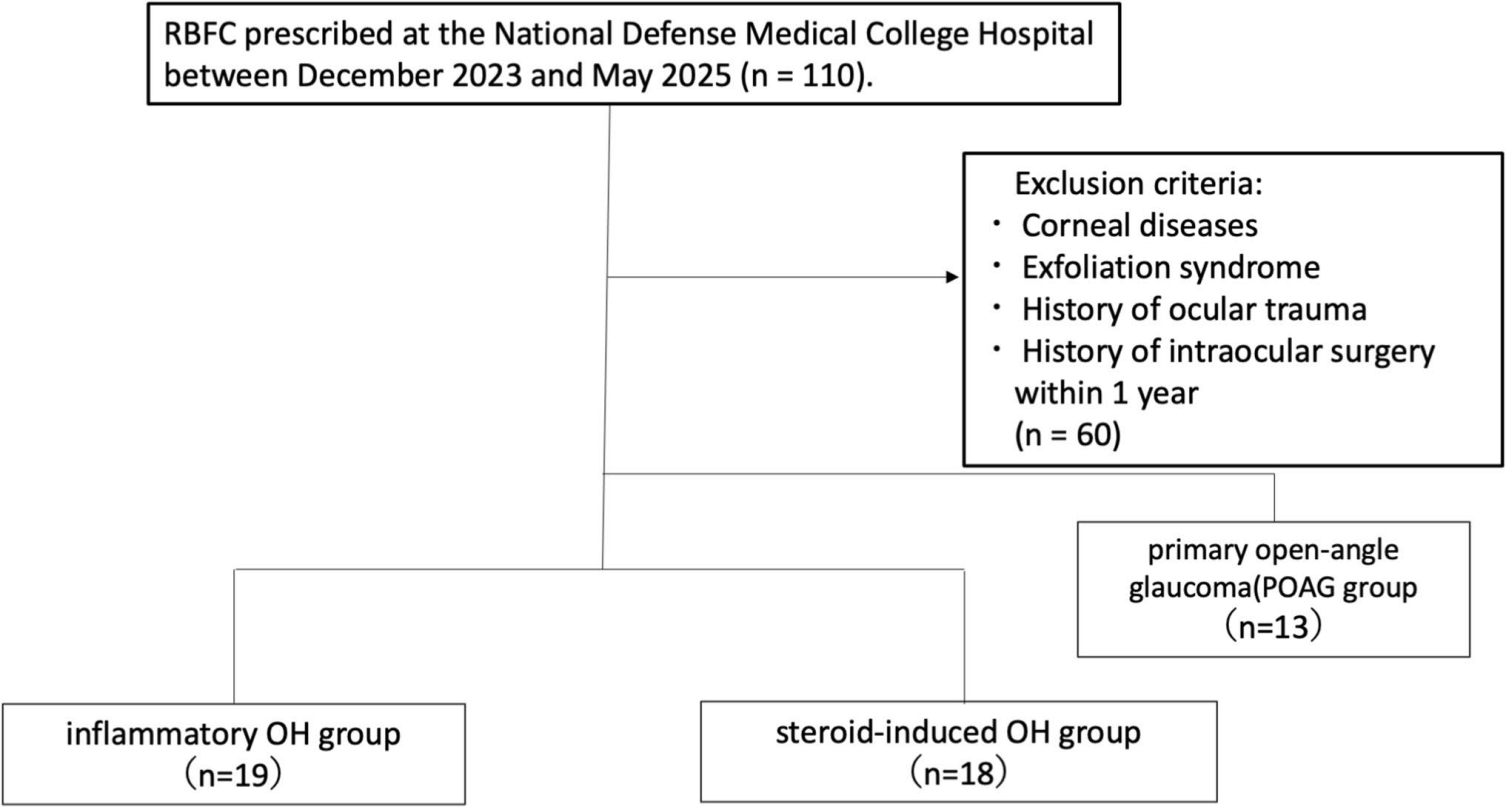
Flowchart illustrating the selection of study participants. Of 110 eyes screened for eligibility, 60 were excluded due to corneal disease, exfoliation syndrome, ocular trauma, or intraocular surgery within one year prior to ripasudil-brimonidine fixed-dose combination (RBFC) initiation. The remaining 50 eyes were included and categorized into inflammatory ocular hypertension (Ocular hypertension, OH; n = 19), steroid-induced OH (n = 18), and primary open-angle glaucoma (primary open angle glaucoma *POAG*; n = 13)

This retrospective study included both uveitic and non-uveitic patients with POAG who were prescribed RBFC at the National Defense Medical College Hospital between December 2023 and May 2025. Inflammatory OH was defined as IOP elevation occurring during active uveitis, in cases without recent corticosteroid initiation or dose escalation, or during steroid tapering, and demonstrating a temporal association with worsening intraocular inflammation documented in the medical records. Steroid-induced OH was defined as IOP elevation occurring after corticosteroid initiation or dose escalation in the absence of active intraocular inflammation. In cases where both inflammatory activity and corticosteroid use were considered potential contributors, classification was determined by consensus among three uveitis specialists after retrospective review of the clinical course, including medication history, inflammatory findings, and longitudinal IOP trends. POAG was included as a clinically well-defined and relatively homogeneous glaucoma subtype with sufficient case numbers for comparison. POAG was diagnosed based on characteristic glaucomatous optic nerve head changes with corresponding reproducible visual field defects, open anterior chamber angles confirmed by gonioscopy, and the absence of secondary causes of glaucoma. Other glaucoma subtypes (e.g. exfoliation glaucoma and other secondary glaucomas) were excluded due to limited sample size and greater pathophysiological heterogeneity, which could further confound interpretation in this retrospective cohort. A flowchart illustrating patient inclusion and exclusion is shown in Figure 1. A total of 110 eyes were screened, and 60 eyes were excluded due to corneal disease, exfoliation syndrome, ocular trauma, or intraocular surgery performed within one year prior to RBFC initiation. The remaining 50 eyes (inflammatory OH; n=19, steroid-induced OH; n=18, and POAG; n=13) were enrolled in the study. This study was conducted in accordance with the principles of the Declaration of Helsinki and approved by the Institutional Review Board of the National Defense Medical College Hospital (IRB approval number: 4970). Given the retrospective design, the need for written informed consent was waived, and patient consent was obtained through an opt-out process via the hospital website.

Demographics at baseline (just before RBFC treatment), treatment duration with RBFC, and IOP measured using Goldmann applanation tonometry, logMAR visual acuity (VA), and anti-glaucoma medication score (1 point per anti-glaucoma topical agent, 2 points for oral acetazolamide) at baseline, the first follow-up (4–6 weeks), and the final observation were analyzed. The final observation was defined as the last follow-up visit for eyes that continued RBFC treatment, and the visit immediately prior to discontinuation for eyes in which RBFC was discontinued. For bilateral cases, the right eye was analyzed.

Statistical analyses were performed with JMP Pro version 17. Repeated measurements of IOP and medication score within each group (baseline, first follow-up, and final visit) were analyzed using repeated-measures analysis of variance (ANOVA) followed by appropriate post hoc multiple comparison tests. Intergroup comparisons were made with the Kruskal–Wallis test, and Steel–Dwass post hoc analysis was applied when significance was detected. A P value < 0.05 was considered statistically significant.

## Results

### Underlying etiologies of uveitis and classification of OH

The distribution of underlying diseases among patients with uveitis and the classification of OH are summarized in Table 1. The most frequent etiology of uveitis overall was herpetic anterior uveitis, followed by sarcoidosis, Tubulointerstitial nephritis and uveitis (TINU) syndrome, and Behçet’s disease. When stratified by the type of OH, inflammatory OH was most commonly observed in eyes with herpetic anterior uveitis, sarcoidosis, and Posner Schlossman syndrome. By contrast, steroid-induced OH was frequently encountered in eyes with sarcoidosis, TINU syndrome, herpetic anterior uveitis, and Behçet’s disease. This classification enabled us to clearly separate eyes in which OH was inflammation-driven from those in which corticosteroid therapy was the predominant trigger.

**Table 1:** Causative diseases and OH classification in patients with uveitis

Causative disease	Inflammatory OH (n = 19)	Steroid-induced OH (n = 18)	Total (n = 37)
Herpetic anterior uveitis	11	3	14
Sarcoidosis	1	5	6
TINU syndrome	0	3	3
Behçet disease	0	2	2
Vogt-Koyanagi-Harada disease	0	1	1
Posner Schlossman syndrome	1	0	1
Acute anterior uveitis	0	1	1
Unidentified uveitis	6	3	9
Total	19	18	37

*OH* Ocular hypertension, *TINU* Tubulointerstitial nephritis and uveitis

### Comparison of patient backgrounds across groups

Baseline demographic and clinical characteristics of patients in the inflammatory OH group, the steroid-induced OH group, and the POAG group are presented in Table 2. Patients in the steroid-induced OH group were generally younger (48.5 ± 21.8 years) and exhibited higher baseline IOP (31.0 ± 7.5 mmHg) compared with the inflammatory OH group (59.6 ± 13.9 years and 28.8 ± 9.9 mmHg). By contrast, the POAG group consisted of significantly older patients (67.8 ± 16.7 years) with lower baseline IOP (mean 15.4 ± 5.72 mmHg). Sex and logMAR VA at baseline were broadly comparable across the three groups. Anti-glaucoma medication score was significantly higher in the POAG group compared with the inflammatory and steroid-induced OH groups. The mean follow-up duration after RBFC initiation was 24.9 ± 29.7 weeks overall, 19.3 ± 24.8 weeks in the inflammatory OH group, 22.5 ± 28.8 weeks in the steroid-induced OH group, and 36.4 ± 36.1 weeks in the POAG group, with no significant differences among the groups. Thus, while the inflammatory and steroid-induced groups were comparable in age and IOP at baseline, the POAG group represented an older population with lower baseline IOP.

**Table 2:** Backgrounds of inflammatory OH, steroid-induced OH, and POAG groups

	Total (n = 50)	Inflammatory OH (n = 19)	Steroid-induced OH (n = 18)	POAG (n = 13)	*P* value
Male/Female	25/25	11/8	8/10	6/7	0.68
Age (years)	57.7 ± 19.1	59.6 ± 13.9	48.5 ± 21.8	67.8 ± 16.7	0.015
Visual acuity at baseline (logMAR)	0.26 ± 0.60	0.37 ± 0.45	0.27 ± 0.86	0.053 ± 0.21	0.37
IOP at baseline (mmHg)	26.1 ± 10.2	28.8 ± 9.9	31.0 ± 7.5	15.4 ± 5.72	<.0001
Medication score at baseline	1.8 ± 1.9	1.6 ± 1.8	1.0 ± 1.7	3.2 ± 1.4	0.0025
Follow-up duration after RBFC initiation (weeks)	24.9 ± 29.7	19.3 ± 24.8	22.5 ± 28.8	36.4 ± 36.1	0.26

*OH* Ocular hypertension, *POAG* Primary open angle glaucoma, *IOP* Intraocular pressure

### Changes in IOP and medication score in the inflammatory OH group with RBFC therapy

**Fig 2.**
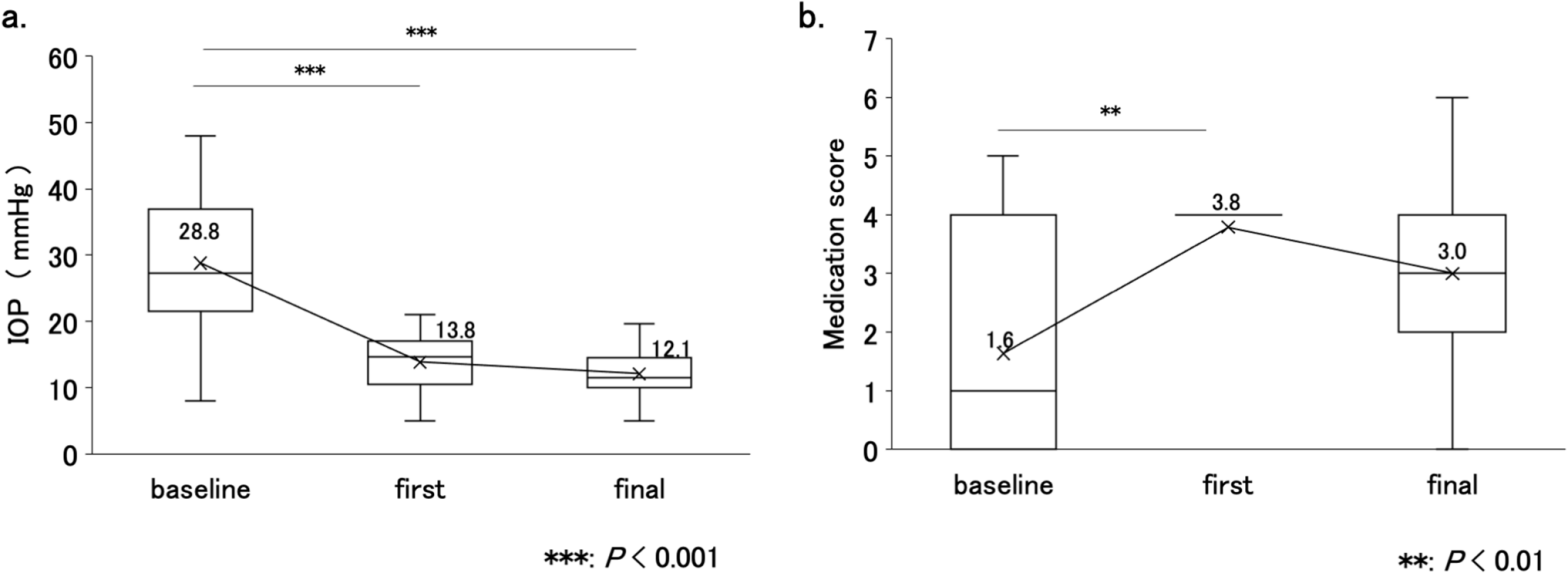
Changes in intra ocular pressure (IOP) and medication score in the Inflammatory OH Group. IOP (**a**) and medication scores (**b**) at baseline (before RBFC initiation), at the first follow-up visit (4–6 weeks after initiation), and at the final visit in the inflammatory OH group are shown. **a** IOP decreased significantly immediately after the initiation of RBFC and remained reduced until the final observation. **b** The medication score increased significantly immediately after RBFC initiation compared with baseline, but decreased by the final observation, showing no significant difference from baseline

Figure 2 exhibits IOP and medication score in the inflammatory OH Group at baseline and after RBFC therapy. IOP decreased significantly following the initiation of RBFC (Fig. 2a), from a mean baseline of 28.8 mmHg to 13.8 mmHg at the first follow-up, and further to 12.1 mmHg at the final observation, indicating that this reduction was maintained throughout the follow-up period. Regarding medication use (Fig. 2b), the mean medication score increased from 1.6 at baseline to 3.8 at the first follow-up, but decreased to 3.0 at the final observation. Since there was no significant difference compared with baseline, this suggests stable IOP control without the need for additional antiglaucoma medications.

### Changes in IOP and medication score in the steroid-induced OH group with RBFC therapy

**Fig 3.**
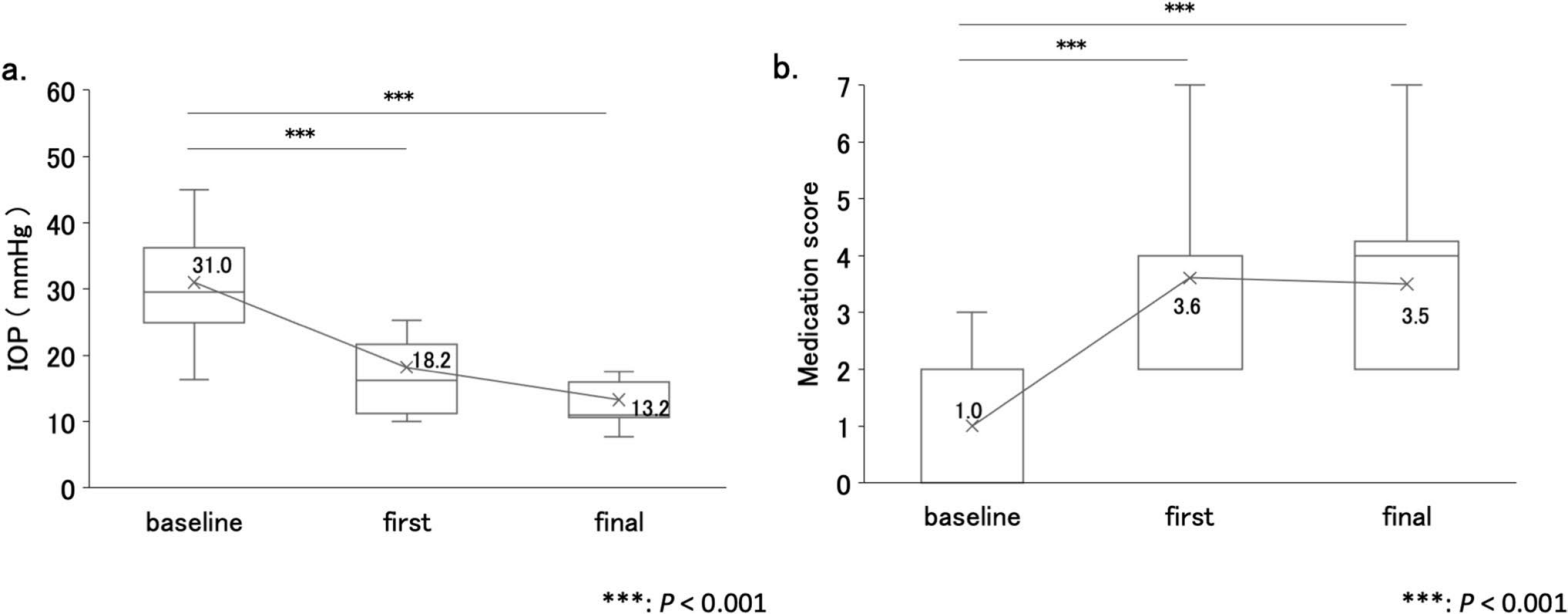
Changes in IOP and medication score in the steroid-induced OH group. IOP (**a**) and medication scores (**b**) at baseline (before RBFC initiation), at the first follow-up visit (4–6 weeks after initiation), and at the final visit in the steroid-induced OH group are shown. **a** IOP decreased significantly immediately after RBFC initiation and continued to decrease further during follow-up. **b** The medication score increased significantly immediately after RBFC initiation compared with baseline and remained unchanged thereafter

Similarly, in the steroid-induced OH group (Fig. 3), IOP decreased significantly following the initiation of RBFC (Fig. 3a), from a mean baseline of 31.0 mmHg to 18.2 mmHg at the first follow-up, and further to 13.2 mmHg at the final observation, indicating that this reduction was maintained throughout the follow-up period. Regarding medication use (Fig. 3b), the mean medication score increased from 1.0 at baseline to 3.6 at the first follow-up, and was maintained to 3.5 at the final observation. This result also indicates stable IOP control without the need for additional antiglaucoma medications in the steroid-induced OH group.

### Changes in IOP and medication score in the POAG group with RBFC therapy

**Fig 4.**
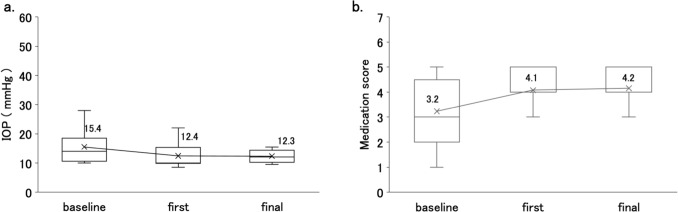
Changes in IOP and medication score in the POAG group. IOP (**a**) and medication scores (**b**) at baseline (before RBFC initiation), at the first follow-up visit (4–6 weeks after initiation), and at the final visit in the POAG group are shown. **a** IOP showed a downward trend after RBFC initiation but without statistical significance. **b** The medication score increased after RBFC initiation and remained unchanged thereafter, with no significant differences compared with baseline

In the POAG group (Fig. 4), IOP showed a modest reduction after RBFC initiation, from 15.5 mmHg at baseline to 12.4 mmHg at the first follow-up and 12.3 mmHg at the final observation (Fig. 4a), however, there was no significant difference. The mean medication score increased from 3.2 at baseline to 4.1 at the first follow-up, and remained unchanged thereafter, with no significant differences compared to baseline (Fig. 4b).

### Changes in visual acuity across the three groups

**Fig 5.**
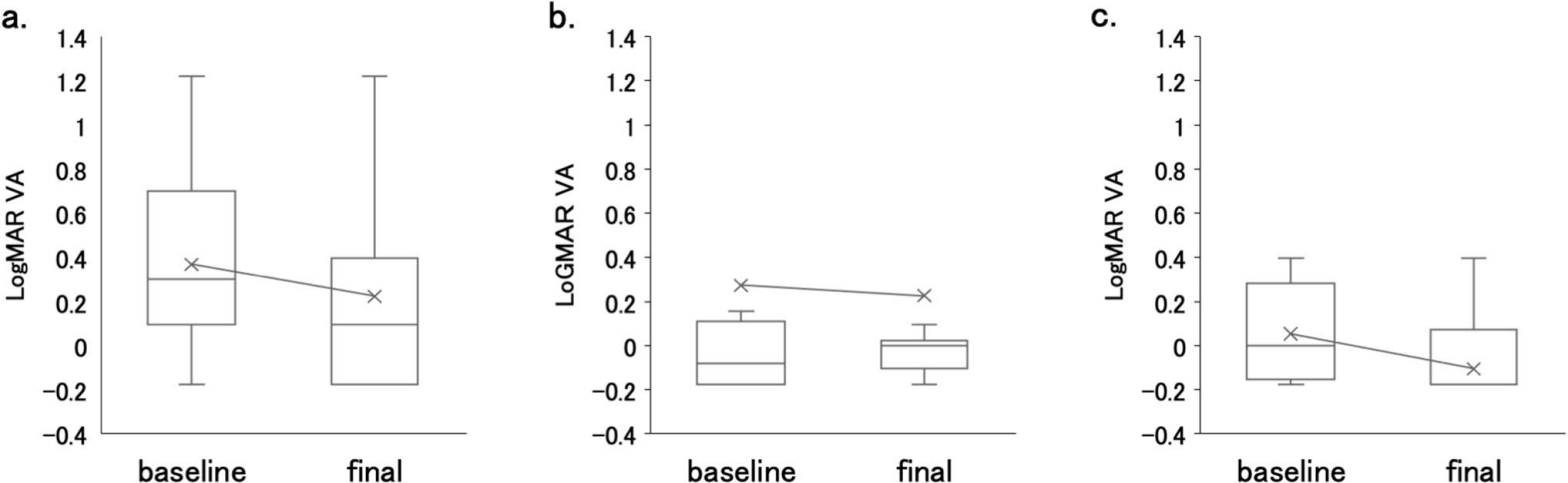
Changes in visual acuity across the three groups. There were no statistically significant decreases in logMAR VA after RBFC treatment in the inflammatory OH (**a**), steroid-induced OH (**b**), and POAG groups (**c**)

In visual function (Fig. 5), logMAR VA showed no statistically significant changes before and after RBFC administration in any of the three groups—inflammatory OH, steroid-induced OH, or POAG. However, a slight improvement in VA was observed across all groups, although the differences did not reach statistical significance. This finding indicates that RBFC therapy is not associated with deterioration in visual acuity during the observation period.

One patient in the POAG group developed mild conjunctivitis approximately 31 weeks after RBFC initiation, leading to discontinuation of RBFC. No cases of blepharitis, allergic conjunctivitis, or uveitis exacerbation requiring treatment escalation were identified in the medical records, and no serious ocular adverse events were observed.

## Discussion

This study demonstrates that fixed-dose RBFC provides significant and sustained IOP reduction in eyes with inflammatory and steroid-induced OH, while maintaining stable visual acuity without causing uveitis recurrence. These results may support the use of RBFC as a therapeutic option in secondary glaucoma associated with uveitis, a population in which treatment is often complicated by both inflammation and corticosteroid exposure.

For reducing IOP in OH secondary to uveitis, since anti-inflammatory and mydriatic eye drops had been usually administered for resolving ocular inflammation and for managing complications, antiglaucoma medications are additionally provided. In many cases, a single agent is insufficient; two or more agents may be required to achieve adequate control [15]. As the number of drops increases, adherence declines [24], and this becomes particularly evident when three or more drugs are required for IOP control. Therefore, fixed-combination therapy holds significant clinical value, as it can improve adherence while minimizing ocular surface toxicity associated with multiple preserved eye drops.

It should be noted that the baseline intraocular pressure (IOP) was lower in the POAG group than in the inflammatory and steroid-induced ocular hypertension (OH) groups. Because absolute IOP reduction is generally greater in eyes with higher baseline IOP, the larger reductions observed in the uveitis-associated OH groups in the present study may partly reflect this baseline difference rather than a true difference in treatment efficacy. Furthermore, since this retrospective analysis did not include baseline-adjusted or percentage IOP reduction analyses, intergroup comparisons to infer differences in RBFC efficacy among glaucoma subtypes were not conducted.

Ripasudil is reported to lower IOP effectively in patients with uveitic glaucoma and steroid responders [14–16]. Our previous study compared inflammatory and corticosteroid-induced OH and demonstrates that ripasudil reduced IOP by approximately 7–10 mmHg from baseline in both groups, with no significant difference between etiologies [15]. Kusuhara et al. also confirm the efficacy of ripasudil in uveitic glaucoma, reporting a mean IOP reduction of approximately 6–7 mmHg (baseline 27.5 ± 7.4 mmHg decreased to 20.6 ± 6.8 mmHg at 1 month and 21.0 ± 6.4 mmHg at 3 months) [16]. In addition, Futakuchi et al. evaluated patients with uveitic, exfoliation, and steroid-induced glaucoma and found mean IOP reductions of about 4–6 mmHg from baseline after ripasudil monotherapy [14], demonstrating comparable pressure-lowering efficacy across different secondary glaucoma subtypes and supporting the reproducibility and consistency of ripasudil’s therapeutic effect observed in the present study. On the other hand, in our current cohort treated with the fixed-dose RBFC, IOP reduction was markedly greater approximately 15–17 mmHg from baseline. This effect substantially exceeded the pressure-lowering magnitude reported for ripasudil alone in previous studies, suggesting a clear additive or synergistic benefit from combining ripasudil with brimonidine.

Brimonidine confers neuroprotection through modulation of glutamate toxicity and activation of trophic factor pathways [19, 21], while ripasudil has also been shown to exert neuroprotective effects on retinal ganglion cells (RGCs) via antioxidative mechanisms [25]. These effects may be particularly relevant in inflamed eyes at risk of optic nerve damage. Therefore, the fixed-dose combination simultaneously targets trabecular outflow, aqueous suppression, and uveoscleral outflow, while potentially reducing inflammation-related tissue injury.

Although brimonidine tartrate is generally well tolerated, several reports describe the occurrence of brimonidine-associated granulomatous anterior uveitis as a rare but recognized adverse reaction [26–29]. In these cases, inflammation typically appeared weeks to months after initiation and was characterized by conjunctival ciliary injection, mutton fat keratic precipitates, and anterior chamber cells, resolving upon discontinuation of the drug. However, in our present study, no cases of uveitis exacerbation or new-onset intraocular inflammation were observed during RBFC therapy. This suggests that RBFC can be safely administered without triggering inflammatory recurrence.

This study is limited by its retrospective design, modest sample size, potential selection bias introduced by exclusion criteria, relatively short follow-up period, intraocular inflammation assessed based on routine clinical documentation, and mild or transient adverse events that may have been unreported. In addition, baseline IOP and age differences among groups and fluctuations in inflammatory activity, including possible steroid adjustments, may have influenced IOP changes, limiting causal interpretation of the observed reductions. The absence of long-term inflammatory outcome measures also prevents conclusions regarding RBFC’s role in modulating intraocular inflammation.

In summary, RBFC provided robust and sustained IOP reduction in both inflammatory and steroid-induced OH, without compromising visual acuity or requiring escalation of glaucoma medications. Compared with earlier studies of ripasudil monotherapy, our results suggest a stronger pressure-lowering effect, highlighting the synergistic benefit of combining ripasudil with brimonidine. RBFC, therefore, represents a promising therapeutic option for secondary glaucoma associated with uveitis, addressing both efficacy and adherence challenges. However, because of the retrospective, uncontrolled design and potential confounding factors such as baseline IOP differences, fluctuations in inflammatory activity, and concurrent steroid adjustments, causal effects cannot be determined. Future prospective studies with larger cohorts and longer follow-up are warranted to confirm these findings and to clarify RBFC’s potential role in inflammation modulation.

## Data Availability

The data that support the findings of this study are available from the corresponding author upon reasonable request.
